# Miscellaneous

**Published:** 1887-03

**Authors:** 


### MISCELLANEOUS.

LIFE WITH A BROKEN NECK.—The *Chicago Journal* tells the story of a remarkable case of fracture of the spine and cord. Mr. Andrew Hamilton was "coaching" some college men in the gymnasium, and, while showing some simple performance on a low crossbar, dropped about four or five feet on the mattress. By some peculiar wrenching of the neck he cracked the fifth cervical bone and compressed the spinal cord. From that moment on the whole of the body below the neck was completely insensible ; nor could he move a single muscle, except to contract two fingers on each hand. He had to call the attendant to open the hand. He was kept alive on milk, which was poured down his mouth, and his body was supported by floating it on a rubber sheet in a tub of water. His mind was perfectly clear ; he talked, read the daily papers, and even consulted his professsor on reading a mathematical work. This continued for fourteen days. He then broke down and died on the sixteenth day after the fall. It seemed hardly possible that life should continue, and the brain go on acting, if the cord were severed, and yet the post mortem examination showed plainly that the cord was reduced to a mass of pus.

HEADACHE IN SCHOOL CHILDREN.—Professor N. J. Bystroff has examined 7,478 boys and girls in the St. Petersburg schools during the last five years, and found headache in 868 ; that is 11.6 per cent. He states that the percentage of headache increases almost in a direct progression with the age of the children, as well as the number of hours occupied by them for mental labor ; thus while headache occurred in only 5 per cent. of the children aged 8, it attacked from 28 to 40 per cent. of the pupils aged 14 to 18. The author argues that an essential obstinate headache in school children is the excessive mental strain enforced by the present educational programme, which leaves out of consideration the peculiarities of the child's nature, and the elementary principles of hygiene. The overstrain brings about an increased irritability of the brain and consecutive disturbances in the cerebral circulation. Professor Bystroff emphatically insists on the imperative necessity for permanently admitting medical men to conferences of School Boards. Of palliative measures he mentions methodical gymnastics, mild aperients in well-nourished children, steel in the anæmic, bromides, inhalation of oxygen, and in severe cases, a temporary discontinuance of all studies.

UNEARTHING TREASURE.—Last spring, Mr. John Dewberry, the keeper of a saloon, in Louisville, Ky., died. Soon after the event, Mrs. Dewberry sold the saloon and moved her residence. On the 9th of November, says a dispatch from Louisville, the new proprietors were called upon shortly after 7 o'clock, A. M., by a woman heavily veiled, who asked to be allowed to go into the back yard. Her request being granted, she went to the left-hand corner of the yard and scraped aside a lot of rubbish, then took a small flower spade she had concealed in the folds of her dress, and dug away the earth until she found a cigar-box. This she removed from the earth and fainted away. The men rushed to the woman's side and found her to be Mrs. Dewberry. The box by her side contained a saltbag full of gold. This they counted, and found twelve twenty-dollar gold pieces, and a number of smaller coins, making a total of $250. When Mrs. Dewberry revived, she declared that while sleeping in her room the night before, she was awakened by the touch of a hand on her forehead. Turning in the dim light, she saw the face of her deceased husband. He stood by the bed, and directed Mrs. Dewberry to the spot where she found the money, and then disappeared. The dispatch says that the story is corroborated by so many reputable gentlemen that it cannot be doubted.—*Banner of Light.*

In 1883, compulsory vaccination was abolished in Zurich, Switzerland. In 1882, there were no deaths from small-pox; in 1883, in 1,000 deaths, two were from small-pox; in 1884, there were three in 1,000; in 1885, there were seventeen in 1,000, and in the first quarter of 1886, there were eighty-five in 1,000. These facts are worthy of very serious consideration.

Two years ago, Miss Maggie Beadling, aged seventeen years, living with her parents in the suburbs of Pittsburgh, Pa., fell down a flight of stairs and injured her spine so severely as to permanently destroy the use of her limbs. Her head was not bruised or hurt in any way. In a few hours after the accident, she fell into a cataleptic state, in which she has remained ever since, with but two lucid intervals, each lasting less than a minute. One of the most wonderful features of her strange case is that she now weighs about one hundred and twenty-five pounds, only five pounds less than when she was hurt two years ago. In all that time she has tasted nothing but milk toast and chicken broth, never over four ounces a day, and has even gone for three days at a time without a morsel of food. She sings almost all the time, many of the airs being new and very beautiful. In one of her lucid intervals, she said she was surrounded by angels. The case is a great puzzle to the doctors.—*R. P. Journal.*

### RIGHT AND DUTY.

If a work you have to do,
Truth to utter, fear dispel;
Do it bravely, do it well:
Heaven asks no more of you.

Though your path in life be drear,
Leave undone no 'lotted task,
Feign no virtue, wear no mask,
God is truth, and heaven is near.

